# Spices for Targeting the Gut Microbiome to Improve Cardiometabolic Health

**DOI:** 10.1093/nutrit/nuag028

**Published:** 2026-05-26

**Authors:** Kristi M Crowe-White, Katelyn E Senkus, Janie C DiNatale

**Affiliations:** Department of Human Nutrition, The University of Alabama, Tuscaloosa, AL, 35401, United States; USDA/ARS Children’s Nutrition Research Center, Department of Pediatrics, Baylor College of Medicine, Houston, TX, 77030, United States; Department of Human Nutrition, The University of Alabama, Tuscaloosa, AL, 35401, United States

**Keywords:** spices, cinnamon, ginger, cardiometabolic, gut microbiome, short-chain fatty acids

## Abstract

The intricate balance between the gut microbial ecosystem and cardiometabolic health underscores the importance of cultivating a healthy gut microbiome. A noteworthy strategy for harnessing the potential of this ecosystem is the consumption of prebiotics—non-digestible compounds promoting the growth of beneficial bacteria. While dietary polyphenols are emerging as significant modulators of microbial proliferation and systemic therapeutic effects, the prebiotic functionality of spice polyphenols remains understudied. It is, however, hypothesized that metabolism of spice polyphenols by intrinsic microflora in the colon may elicit downstream cardiometabolic effects resulting from the stimulated release of gut-derived metabolites, specifically short-chain fatty acids. This brief article highlights research on the functionality of spices, namely cinnamon and ginger, to influence microbial proliferation, the production of gut-derived metabolites and hormones, and prospective health outcomes.

## INTRODUCTION

Microbial regulation of host metabolism is significantly impacted by the composition of the diet. For example, when consumption of undigested or minimally digested substrates (ie, dietary fiber) reach the colon, gut microbes hydrolyze and may ferment these compounds for utilization as energy to foster proliferation and functionality.^1^ Similar to dietary fiber, polyphenols are metabolized primarily in the colon, with 90% to 95% of dietary intake evading digestion in the upper gastrointestinal tract.^2^ While the biological functionality and impact of polyphenols is not solely attributed to colonic metabolism, the degree of metabolism in the colon elicits specific changes in the composition and/or activity of the gastrointestinal microbiota, thus conferring benefits to the host.^3^ To date, there is mounting evidence that polyphenols impart cardiometabolic benefits as a result of the host–microbe interplay, which is the focus of this research.^4^ Nevertheless, our knowledge of the role of spice polyphenols as prebiotics is sparse.

Among the numerous sources of dietary polyphenols, spices are the most concentrated food source of polyphenols with established roles in gut microbial modulation.^5^^,^^6^ One of the primary mechanisms of action of spice polyphenols is the result of bacterial metabolism and the production of metabolites such as short-chain fatty acids (SCFAs)—acetate, propionate, and butyrate. These gut-derived metabolites elicit downstream effects both directly and indirectly on lipid metabolism, glucose homeostasis, inflammation, gut hormone release, and cardiovascular health.^7–10^ In addition to the aforementioned cardiometabolic influences, SCFAs serve as fuel for colonocytes and as signaling molecules in functional pathways, including the gut–brain axis.^11^ Such bidirectional interactions underscore the importance of cultivating a healthy gut microbiome.^12^ As diet is among the strongest determinants of gut microbial composition and function, the prebiotic functionality of spices rich in polyphenols may represent a flavorful vehicle for cultivating a healthy gut microbiota and influencing overall cardiometabolic health.^13^^,^^14^ However, there is minimal research to suggest which spices would optimally enhance gut functionality; furthermore, once identified, the amount of intake to confer these benefits has yet to be elucidated.

In an effort to advance the understanding of the health benefits of spices and herbs beyond being antioxidants and immunomodulators, this brief aims to present the latest insights into leveraging the power of spice polyphenols in their effects on the gut microbiota and the prospective health outcomes from gut-derived metabolites, while highlighting the feasibility of a clinical trial investigating the effect of a culinary dose of spices on gut microbiota, gut-derived metabolites, and cardiometabolic outcomes (ClinicalTrials.gov #NCT06313580).^15^ Herein, the influence of cinnamon (*Cinnamomum)* (3 g) and ginger (Zingiberaceae*)* (1 g) incorporated into yogurt—the standard of care for gut health—are reviewed.^16^ Acknowledging the previously documented prebiotic effect of polyphenols and probiotic effects of yogurt, this research highlights a pre/pro cocktail or synbiotic food-first intervention to enhance gut health and influence cardiometabolic health.^17^

## GUT MICROBIOME AND GUT-DERIVED METABOLITES

Spices and herbs may modulate microbial communities through selective metabolism of polyphenols, thus fostering microbial proliferation and the production of gut-derived metabolites.^6^ Previously funded research suggests that cinnamon and ginger exhibit a prebiotic effect by stimulating the proliferation of commensal bacteria in the colon, thereby promoting gut health.^14^ Our work corroborates these findings, with daily consumption of cinnamon and ginger resulting in beneficial effects on the gut microbial population, such that the addition of spices to yogurt in the diet induced significant changes in the SCFA-producing bacteria, including increases in *Alistipes*, *Roseburia,* and Ruminococcaceae. As a result of this proliferation, enhancement in SCFA production was observed, with acetate increasing by 24% and butyrate by 19%. These findings advance our understanding of the selective enrichment of microflora with known health benefits through the intake of the spices cinnamon and ginger.

It is well established that inter-individual variation exists in polyphenol bioavailability and metabolism.^18^ However, when grouped as responders and non-responders, those among whom enrichment of SCFA-producing taxa were observed exhibited an increase in the gut-derived hormone peptide tyrosine-tyrosine (PYY) at the magnitude of 7.5%. The ability of gut microbiota to influence PYY secretion results from SCFA signaling, particularly butyrate in a dose-dependent manner.^19^ Our results suggest that increases in fecal SCFAs were in the same direction, as this gut-derived hormone is involved in the regulation of food intake and satiety.

## CARDIOMETABOLIC OUTCOMES

Previous research suggests that SCFAs play a regulatory role in glucose homeostasis by stimulating secretion of PYY as well as glucagon-like peptide-1 (GLP-1), which enhances the uptake of glucose into muscle and adipose tissue.^9^ Among the serum biomarkers assessed in our research, serum glucose was significantly inversely correlated with butyrate and propionate.

Additional cardiometabolic outcomes influenced by SCFAs include stimulation of leptin release, involved in satiety and energy expenditure and modulation of cholesterol metabolism. For example, intake of cinnamon and ginger in yogurt after 4 weeks resulted in significant increases in serum leptin upwards of 30%. While adipose tissue remains the primary driver for leptin synthesis, SCFAs have been shown to stimulate leptin expression in adipocytes through activation of free fatty acid receptor 3 (FFAR3), which preferentially binds to butyrate and propionate for activation.^20^ Thus, as prebiotics stimulate the production of SCFAs, the prebiotic nature of spice polyphenols may indirectly influence circulating leptin levels. Additionally, SCFA activation of the FFAR3 pathway plays a key regulatory role in lipid metabolism, specifically hepatic lipid metabolism.^9^^,^^21^ As such, spice polyphenols may influence cholesterol metabolism. Using the total cholesterol : high-density-lipoprotein cholesterol ratio (a robust clinical predictor of future cardiac events),^22^ our previously published findings suggest that consumption of cinnamon and ginger daily decreased the ratio by approximately 4.4%, thus reducing lipid-associated cardiovascular risk factors.

Linking the intake of spice polyphenols with both gut microbial functionality and cardiometabolic health is a foundational step in understanding the magnitude of the effect that spices have on human health. Given that diet rapidly and reproducibly influences the gut microbiome, it should be acknowledged that harnessing the power of the native microbial flora can result in significant downstream effects.^23^ Additionally, the integration of spice polyphenols with prebiotic functionality and probiotics from yogurt may assist in leveraging the intricate interplay between microbial communities and human physiology. The results of this study suggest that spices, namely cinnamon and ginger, added to yogurt represent a palatable synbiotic, food-first intervention for conferring such benefits.

## CONCLUSIONS

Although the microbial-mediated influence of spices on gut-derived metabolites and cardiometabolic health are not fully elucidated, our research has shown that polyphenol-rich spices such as cinnamon and ginger augment the release of these metabolites, resulting in significant health implications after 4 weeks; moreover, these findings support the role of spices in enhancing the benefit of yogurt as the traditional standard of care for gut health by stimulating the proliferation of SCFA-producing bacteria. Taken collectively, fueling the gut through spice ingestion represents a palatable, cost-effective, and therapeutic approach for bolstering cardiometabolic health.
